# Eosinophilic crystalline pneumonia, an age-related lesion in mice

**DOI:** 10.31491/apt.2020.12.047

**Published:** 2020

**Authors:** Jenna J Klug, Jessica M Snyder

**Affiliations:** a Department of Comparative Medicine, School of Medicine, University of Washington, Seattle, WA, USA.

**Keywords:** Epithelial hyalinosis, crystals, acidophilic macrophage pneumonia (AMP), eosinophilic crystalline pneumonia (ECP)

## Abstract

Eosinophilic crystalline pneumonia (ECP), also known as acidophilic macrophage pneumonia (AMP), is a common intrapulmonary lesion that increases in prevalence with age in mice, especially those on a C57BL/6 and 129Sv background. Gross changes may be evident in severe cases as lobar to diffuse red to brown foci throughout the lungs, which fail to collapse. Definitive diagnosis is by histopathology, which shows the accumulation of brightly eosinophilic crystals within macrophages or free within lumens of alveolar spaces and conducting airways. Granulocytes, multinucleated giant cells, and epithelial hyalinosis may also be present in affected areas of the lung. The disease may represent a cause of morbidity and mortality when other disease processes interfere with clearance, leading to the accumulation of crystals and crystal laden macrophages in airways, resulting in dyspnea. Other anatomic locations may be affected by epithelial hyalinosis and/or crystals as part of the syndrome, including respiratory tract, stomach, gall bladder, bile duct, and pancreatic duct.

Eosinophilic crystalline pneumonia (ECP), also known as acidophilic macrophage pneumonia (AMP), is a common pulmonary lesion that increases in incidence with age in mice [1]. It occurs across most laboratory strains and wild mice, although has a higher prevalence in C57BL/6, 129Sv, Swiss, Ptpn6me motheaten mice, severe combined immunodeficiency (SCID), and various types of genetically engineered mice on a C57BL/6 or 129 background [1, 2]. Gross changes range from multifocal parenchymal infiltrates to lobar to diffuse areas of red and tan discolorations in the lungs, which fail to collapse upon opening the thoracic cavity [1]. Definitive diagnosis is made by histopathology and characterized by an intrapulmonary accumulation of brightly eosinophilic acicellular (needle-shaped) to rectangular crystals [1, 3, 4]. Crystals may be present extracellularly within alveolar spaces and conducting airways (Figure 1, Figure 2C), or within the cytoplasm of macrophages and multinucleated giant cells (Figure 2A, B). Affected areas of lung may also contain granulocytes and epithelial hyalinosis [2, 4].

ECP can occur spontaneously or concurrently with other lung pathology, such as neoplastic, hyperplastic, infectious, hypersensitivity, and lymphoproliferative diseases [1]. In aging mice, the condition may represent a cause of morbidity and mortality when found in association with any disease process that impairs normal clearance of alveolar exudate, causing large numbers of crystal-laden macrophages to accumulate in air spaces, leading to respiratory distress and death [2]. The crystals are composed of chitinase-3-like-3 (CHI3L3) protein (formerly known as YM1) and contain iron, alpha-1 antitrypsin, immunoglobulin, and granulocyte breakdown products [2, 5]. Morphologically, they are similar to Charcot-Leyen crystals, which are present in humans and nonhuman primates with eosinophil-rich diseases such as asthma and helminth infestations [1]. Although the lesions in the lungs are the most overt manifestation of this condition, hyalinosis may also occur at other anatomical locations as part of the syndrome in predisposed mice, including epithelium of olfactory, nasal respiratory, middle ear, trachea, lung, stomach, gall bladder, bile duct and pancreatic ducts [2, 5, 6]. In addition, extracellular crystals may also be present in the glands of these tissues [3].

## Figures and Tables

**Figure 1.**
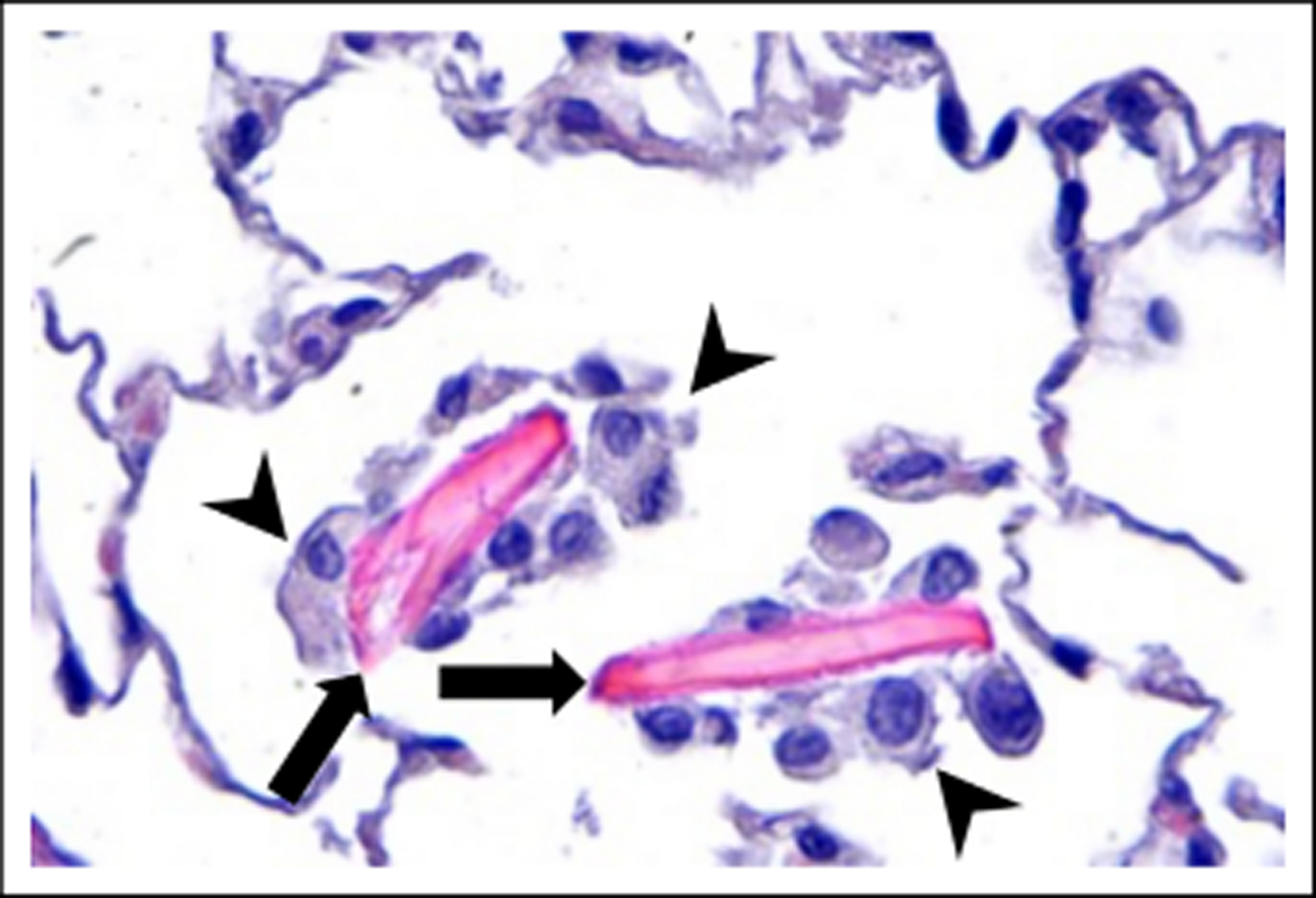
Eosinophilic crystalline pneumonia in a 28-month-old male C57BL/6 mouse. ECP crystals (arrows) in an alveolar sac of the lung. The crystals are large, eosinophilic, rectangular, extracellular, and associated with macrophage infiltrates (arrowheads), 400x, HE.

**Figure 2.**
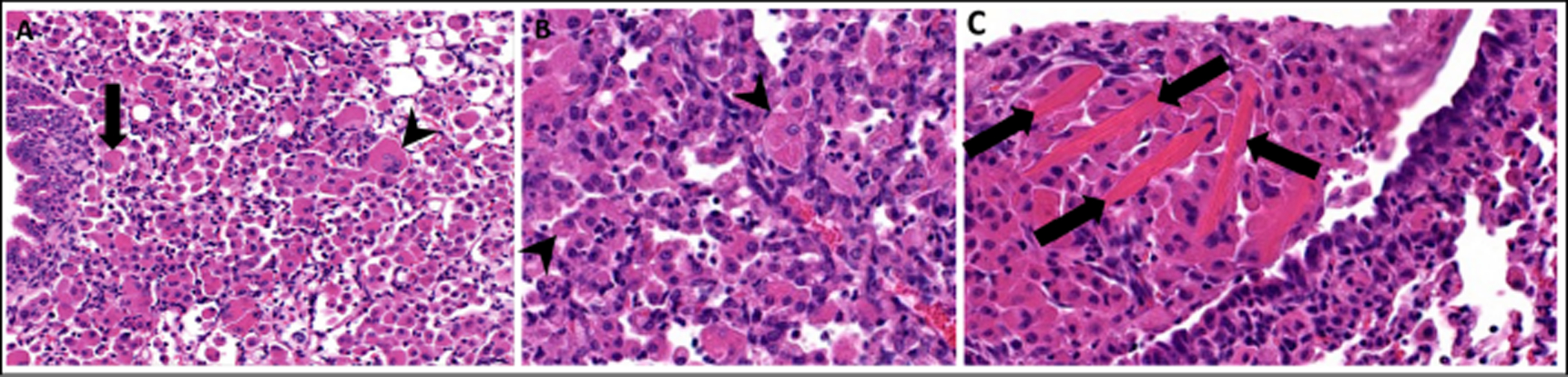
Eosinophilic crystalline pneumonia in a 16-month-old female genetically engineered mouse associated with pulmonary adenocarcinoma. **(A)** Lung moderate to severely affected by ECP. Large numbers of macrophages are present within alveolar spaces, with some multinucleated giant cell formation. Intracellular eosinophilic crystals are present within some macrophages (arrow) and multinucleated giant cells (arrowhead), 200x, HE. **(B)** Higher magnification of ECP demonstrating small, needle-shaped, eosinophilic crystals within the cytoplasm of macrophages (arrowhead), 400x, HE. **(C)** Higher magnification of ECP demonstrating large, rectangular extracellular eosinophilic crystals, (arrows) 400x, HE.
